# Qualitative and Quantitative Comparison of Aromatic Oil Components and Antifungal Effects of *Cymbopogon flexuosus* Obtained with Supercritical CO_2_, Microwave–Ultrasonic, Steam Distillation, and Hydrodistillation Extraction Techniques

**DOI:** 10.3390/molecules28196870

**Published:** 2023-09-29

**Authors:** Nidal Jaradat

**Affiliations:** Department of Pharmacy, Faculty of Medicine and Health Sciences, An-Najah National University, Nablus P.O. Box 7, Palestine; nidaljaradat@najah.edu

**Keywords:** aromatic oils, *Cymbopogon flexuosus*, supercritical CO_2_, microwave–ultrasonic, steam distillation, hydrodistillation, antifungal, GC–MS

## Abstract

*Cymbopogon flexuosus* is a highly valued botanical species with significant applications in the food and food supplement industries, medicine, and cosmetics. The effects of four extraction techniques, supercritical CO_2_, microwave–ultrasonic, steam distillation, and hydrodistillation techniques, on the yield, phytochemical constituents, and antifungal activity against nine fungal species of *Cymbopogon flexuosus* aromatic oil (AO) were explored in this investigation. Gas chromatography connected with a mass spectrometry apparatus was employed for the qualitative and quantitative analyses of the investigated plant AOs. In addition, using the broth microdilution method, minimum inhibitory concentrations (MICs) were calculated for several fungi species. The supercritical CO_2_ method gave the highest yield of AO (11.62 ± 0.03 (*w*/*w*)) followed by the microwave–ultrasonic method (1.55 ± 0.05% (*w*/*w*)) and the steam distillation method (1.24 ± 0.04% (*w*/*w*)), while the hydrodistillation methods gave the lowest yield (1.17 ± 0.01 (*w*/*w*)). In addition, eighteen molecules were specified in the AOs obtained with the supercritical CO_2_, microwave–ultrasonic, steam distillation, and hydrodistillation techniques, which constituted 99.36, 98.6, 98.21, and 98.31% (*v*/*v*) of the total oils, respectively. Additionally, linalyl acetate was the trending molecule in the microwave–ultrasonic and steam distillation methods, representing 24.61 and 24.34% (*v*/*v*), respectively, while geranial was the dominant molecule in the AOs extracted with the hydrodistillation and supercritical CO_2_ extraction techniques (27.01 and 25.6% (*v*/*v*), respectively). The antifungal screening results revealed that the tested *C. flexuosus* AOs have potential antifungal effects against all the screened fungi species. The antifungal effect of the AOs extracted with the steam distillation and microwave–ultrasonic methods was remarkable compared with that of the commercial antifungal drug Fluconazole. However, the AOs extracted with these two methods have a more potent antifungal effect against *Candida parapsilosis* than that of Fluconazole with MICs of 3.13 ± 0.01, 3.13 ± 0.01, and 6.25 ± 0.91 µg/mL, respectively. The same effects were also observed against *Trichophyton rubrum* with MICs of 6.25 ± 0.91 µg/mL, respectively. The results of this investigation demonstrated that the steam distillation and microwave–ultrasonic methods are promising processes for the extraction of *C. flexuosus* AO with a potent antifungal effect. This may be an advantage for the utilization of *C. flexuosus* AO over some antifungal synthetic agents commonly utilized as medicines, preservatives, food additives, cosmetics, and nutrient supplements.

## 1. Introduction

Pathogenic fungi have a significant influence on people’s health all over the world as well as on the biodiversity of the planet and the availability of food. Due to innovations in contemporary medical science, the treatment of a broad range of human diseases has considerably improved, and infectious disease outbreaks have been significantly mitigated as a result, which has increased the median lifespan of humans [1]. Given this, opportunistic fungal infections have become a prominent cause of human mortality, particularly in people who already have underlying health issues or who are receiving immunosuppressive therapies; the number of deaths that may be attributed to these pathogens is estimated to be 1.5 million each year [2]. The most prevalent causes of illness are *Candida, Cryptococcus*, and *Aspergillus* species. Many Candida spp. are endosymbiont types or innocuous commensals of hosts, including humans; nevertheless, when mucosal barriers are breached or the immune system is weakened, they may invade the hosts and cause illness, a condition known as an opportunistic infection [3]. *Candida* species are found on the majority of surfaces of mucosal membranes, including the gastrointestinal system and the skin. *Candida albicans* is a primary cause of nosocomial infections with mortality rates frequently exceeding 40 percent despite therapy [4,5]. It is by far the most frequently isolated fungus species that may lead to thrush or candidiasis infection in animals and humans [6]. Antibacterial medications may increase fungal (yeast) infections, such as gastrointestinal Candida overgrowth and mucosal penetration. Even though women are more vulnerable to vaginal fungal infections, men may be affected as well. Certain variables, such as long-term antibiotic usage, raise the risk for both women and men. Fungal infections are more common in diabetics and immunocompromised individuals [7,8].

Over the last two decades, the *Candida parapsilosis* strain has emerged as a major human pathogen and is now recognized as one of the main causes of aggressive candidal disease. Patients in critical care units and neonates are particularly vulnerable to serious infection [9].

*Candida tropicalis* has risen to prominence as one of the most significant *Candida* species. It is often recognized as the second-most virulent Candida species behind *C. albicans*. In the majority of studies, this species has outperformed *C. albicans* when it comes to biofilm formation. In addition, it produces some additional virulence factors, such as an attachment to epithelial cells and buccal endothelium and the release of lytic enzymes that include proteinases, phospholipase, and hemolysins. Molecular and phenotypic techniques have been used to detect this species, which is highly similar to *Candida albicans*. *C. tropicalis* may be the second or third etiological cause of candidemia in Asian and South American regions [10].

*Candida glabrata* was previously believed to be a nonpathogenic, commensal fungus found in human mucosal tissues and received little attention. In the HIV-infected population, the incidence of systemic and mucosal infections caused by *C. glabrata* has increased significantly due to the increased use of immunosuppressive medications. *C. glabrata* is the second or third most prevalent agent responsible for superficial (oral, esophageal, vaginal, and urinary) or systemic candidal infections, which are frequently nosocomial [11].

Nosocomial infections that are caused by the fungus pathogen *Candida auris* are a major health concern worldwide. It has many of the virulence traits of other invasive Candida infections and also displays multiple drug-resistant patterns to standard anticandida treatments [12].

*Blastomyces dermatitidis* is the fungus that causes blastomycosis and is responsible for the overwhelming majority of symptomatic cases of blastomycosis, including pulmonary disseminated disease that occurs in immunocompromised patients, and up to 10% of these cases involve renal involvement [13].

*Histoplasma capsulatum* is a multifaceted fungus that predominantly affects the respiratory system and creates histoplasmosis. Mold and yeast are the two distinct morphological forms of *H. capsulatum*. It exists in the environment as a fungus with filamentous structures that produce conidia, which are microscopic particles. When inhaled by humans or animals, these particles can induce lung inflammation [14].

Traditional herbal medicines are extremely important due to their vital role in the discovery of new medications [15]. Indeed, natural compounds and their semi-synthetic derivatives have been determined to be sources of therapeutics for the last two centuries. In addition, they provide complex chemical patterns, and their value as physiological modulators has received considerable attention [16,17].

Aromatic oils (AOs), also known as essential oils, have been utilized in a wide range of food, pharmaceutical, and cosmetic applications. Some kinds of AOs produced from herbs were shown to be more effective than commercial antibiotics in inhibiting bacterial and fungal growth. The exact inhibition could be attributed to the active principle in the AO acting singly or in concert [18].

*Cymbopogon* spp. display wide variation in morphological characteristics and AO composition at inter- and intra-species levels, and over the years, different chemo cultivars that vary in their aroma have been selected or bred by crossing with other cultivars or closely related species. The most common economically sound species of the genus *Cymbopogon* viz. are *C. flexuosus*, *C. citratus*, and *C. martinii* var. *motia* [19].

*Cymbopogon flexuosus* (Nees ex Steud.) W.Watson (Poaceae family) is commonly known as Lemongrass, Malabar grass, East-Indian lemon grass, or Cochin grass. It is an edible plant used as a flavoring agent for many types of food and has several medicinal and cosmetic applications. It is an aromatic medicinal grass that is native to America, Africa, and Asia. It is a perennial monocotyledonous herb that can grow up to 6 feet high and 4 feet wide. It has long, slender, drooping, bright green, simple leaves with entire margins. The odor of the plant’s AO makes it useful in many industries, such as perfumes, detergents, and soaps. A large group of traditional medical applications of *C. flexuosus* remains today. It is commonly used for the treatment of bone fractures, headaches, fever, wounds, hypertension, rheumatism, and diabetes [20]. It also has many bioactive compounds, which can be grouped into tannins, saponins, phenols, flavonoids, terpenoids, and alkaloids. The health-restorative capability of *C. flexuosus* AO may be attributed to the different secondary metabolites that it produces [21]. Studies show that *C. flexuosus* possesses different pharmacological properties, including antimycobacterial, antimutagenic, hypoglycemic, antioxidant, antimalarial, anti-inflammatory, anti-amoebic, antibacterial, antifilarial, antidiarrheal, and antifungal properties [22]. These results are very hopeful and show that this herb should be studied more and more to go further with these results and use it properly for other possible therapeutic effects that could be discovered.

*C. flexuosus* is indigenous to India and cultivated on a large scale in China, Indonesia, Madagascar, Haiti, Dominica, Mexico, and Brazil. In fact, India produces approximately 1000 tons of *C. flexuosus* AO per year and exports it to Japan, Australia, Germany, England, and America [19].

The chemical composition of AOs can vary significantly from one region to another due to a variety of factors related to geography, harvesting methods, extraction procedures, climate, seasonal variations, and rainfall. Due to these variations, AOs from the same plant species can have distinct aromas, therapeutic properties, and uses depending on where and how they are cultivated, harvested, and extracted [23].

Hence, AOs have shown promise as antifungal agents in prior in vitro and in vivo studies [24,25,26]. In addition, the extraction processes are considered the main factors that affect the phytochemical constituents of medicinal plants’ AOs and, consequently, their medicinal properties [27].

Therefore, the present investigation aims to investigate the effect of four extraction techniques, supercritical CO_2_, microwave–ultrasonic, steam distillation, and hydrodistillation techniques, on the extraction yields and chemical and antifungal features of *C. flexuosus* AOs from Palestine to find the best technique for applications in the pharmaceutical, cosmeceutical, and food industries.

## 2. Results

### 2.1. Extraction Yields

Table 1 shows the yields of the *C. flexuosus* AOs that were obtained from the hydrodistillation, steam distillation, microwave–ultrasonic, and supercritical CO_2_ extraction procedures. The supercritical CO_2_ method gave the highest yield (1.62 ± 0.03% (*w*/*w*)) followed by the microwave–ultrasonic method (1.55 ± 0.05% (*w*/*w*)) and the steam distillation method (1.24 ± 0.04% (*w*/*w*)), while the hydrodistillation methods gave the lowest yield (1.17 ± 0.01% (*w*/*w*)).

### 2.2. Qualitative and Quantitative Analysis

The characterized compounds of the *C. flexuosus* AOs that were extracted using the four different methods along with their relative retention indices and concentrations are presented in Table 1. The GC–MS analysis outcomes revealed that 18 molecules were quantified and qualified with the supercritical CO_2_, microwave–ultrasonic, steam distillation, and hydrodistillation techniques, representing 99.36, 98.6, 98.21, and 98.31% (*v*/*v*) of the total AO, respectively. Linalyl acetate was the trending molecule in the microwave–ultrasonic and steam distillation methods, representing 24.61 and 24.34% (*v*/*v*), respectively. In addition, geranial was the major compound identified with the hydrodistillation and supercritical fluid extraction techniques (27.01 and 25.6% (*v*/*v*), respectively).

**Figure 1:**
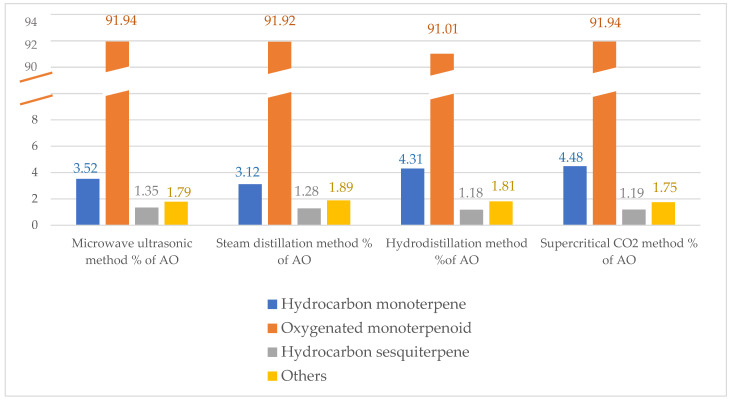
Phytochemical groups (% (*v*/*v*)) in the *C. flexuosus* AOs extracted utilizing hydrodistillation, steam distillation, microwave–ultrasonic, and supercritical CO_2_ extraction methods.

The concentrations of compounds varied with the extraction method. Moreover, according to their chemical functional groups, the identified molecules were classified into three major phytochemical classes: hydrocarbon monoterpenes, oxygenated monoterpenoids, and hydrocarbon sesquiterpenes. Oxygenated monoterpenoids were the most abundant phytochemical group determined in the natural constituents obtained with the supercritical CO_2_, microwave–ultrasonic, steam distillation, and hydrodistillation techniques, representing 91.94, 91.94, 91.92, and 91.01% (*v*/*v*), respectively (Figure 1).

### 2.3. Antifungal Activity

The brain heart infusion broth microdilution assay revealed that the AOs from *C. flexuosus* herb extracted via the hydrodistillation, steam distillation, microwave–ultrasonic, and supercritical fluid extraction techniques have potential antifungal activity against all the screened fungi species, as shown in Table 2. However, compared with the strong antifungal drug Fluconazole, the AOs obtained with the steam distillation and microwave–ultrasonic methods have the most potent antifungal activity against all the tested microorganisms.

## 3. Discussion

Plant substances, including AOs, have been used extensively in traditional medicine and agro-food science to treat a variety of illnesses and extend the shelf life of foods since ancient times [28]. Even in the recent era, plants containing AOs are still widely utilized to cure human diseases and manufacture cosmetics, food additives, and supplements. Due to their ability to efficiently eliminate a variety of bacterial, fungal, and viral diseases, AOs offer enormous promise in the area of medicines [29]. The AOs are efficient against a variety of fungal infectious diseases due to the presence of various oxygenated components. Researchers have lately been motivated to find new antifungal lead molecules to treat a variety of human infections due to the incidence of antifungal medication resistance [30]. Many pathogenic fungi are not inhibited by certain chemical medications that are now on the market. Additionally, due to their potential for environmental hazards, acute toxicity, and carcinogenic consequences, the use of synthetic compounds for the control of pathogenic microorganisms is restricted. In this sense, using AOs to manage multi-drug-resistant pathogenic fungi may help fight a variety of infectious disorders [31]. Therefore, the purpose of the current study was to extract the AO from the aerial portion of the C. flexuosus plant using four different extraction techniques and evaluate their antifungal activity against several species of virulent fungi.

### 3.1. Yields of Extractions

The selection of the process of extraction used for AOs may have a substantial influence on both the quantity of oil obtained and the overall quality of the end product [32]. In our experimental work, the supercritical CO_2_ extraction technique gave the highest *C. flexuosus* AO yield (1.62 ± 0.03% (*w*/*w*)) followed by the microwave–ultrasonic procedure (1.55 ± 0.05% (*w*/*w*)), while the steam and hydrodistillation methods gave the lowest yield (1.24 ± 0.04 and 1.17 ± 0.01% (*w*/*w*), respectively). In general, the extraction conditions used affect the yields of *C. flexuosus* AO. *C. flexusosus* usually yielded 1 to 2% AO on a dry weight basis, which contains mainly citral [33].

This outcome was in agreement with the study of Singh et al. [34], which investigated the effect of the hydrodistillation, solvent extraction, ultrasonic-assisted extraction, and supercritical CO_2_ extraction techniques on the yield of *Eucalyptus globulus* AO.

Our results are also consistent with the study of Danh et al., which investigated the effect of different extraction methods on the yields of *Lavandula angustifolia* AO and found that the AO yield with supercritical CO_2_ extraction techniques was 6.7% (*w*/*w*) compared with that of the hydrodistillation method, which gave a lower yield of 4.6% (*w*/*w*) [35]. In fact, due to the use of a high flow rate of CO_2_ and high pressure, the yield of AO was the highest compared with the other investigated methods. Also, in second place was the microwave–ultrasonic method, which uses microwaves that can penetrate deep into the plant tissue and obtain more oil in addition to ultra-sonication that vibrates the plant material strongly to obtain more AOs. Our experimental results showed that the high CO_2_ flow rate increased the extraction yield within a short duration of time (15 min).

Unlike other extraction methods, namely the organic solvent, microwave–ultrasonic, steam distillation, and water distillation methods, the supercritical CO_2_ extraction procedure leaves no solvent residue behind and is inexpensive. Furthermore, the carrier CO_2_ gas utilized in this procedure is inert, tasteless and odorless, non-flammable, and non-toxic. This stands in contrast to the alternative extraction methods that exhibit lower extraction efficiency, degradation of thermolabile molecules, loss of certain volatile substances, excessive employment of organic solvents, and prolonged time consumption [36].

In addition, it has been demonstrated that this method produces AO with a natural scent that is unaffected by chemical changes induced by heat and water and is devoid of solvent leftovers and objectionable compounds [37].

### 3.2. Phytochemical Qualitative and Quantitative Analysis

Qualitative and quantitative GC–MS analysis showed that 18 compounds were characterized in the *C. flexuosus* AOs obtained with the supercritical CO_2_, microwave–ultrasonic, hydrodistillation, and steam distillation extraction methods, which constituted 99.36, 98.6, 98.31, and 98.21% (*v*/*v*) of the total oils, respectively. However, the GC–MS analysis also revealed that the supercritical CO_2_, microwave–ultrasonic, steam, and hydrodistillation extraction methods produced AOs with different phytochemical components. In point of fact, four major molecules, namely linalool, neral, linalyl acetate, and geranial, were the trend molecules in all investigated AO samples, which accounted for 85.65, 84.57, 84.44, and 84.74% (*v*/*v*), respectively. Among them, geranial was the main compound in the hydrodistilled AO, accounting for 27.01% (*v*/*v*), followed by the supercritical CO_2_, steam distilled, and microwave–ultrasonic extracted AOs, which accounted for 25.6, 20.96, and 20.64% (*v*/*v*), respectively. Moreover, linalyl acetate was the abounded molecule of the AO extracted with microwave–ultrasonic extraction followed by the steam distillation, supercritical CO_2,_ and hydrodistillation extracted AOs, representing 24.34, 20.86, and 18.77% (*v*/*v*), respectively. In addition to that, neral was the major constituent of the AO extracted via the hydrodistillation technique followed by the AOs extracted with the supercritical CO_2_, microwave–ultrasonic, and steam distillation extraction techniques, which accounted for 21.49, 17.41, and 16.93% (*v*/*v*), respectively. Moreover, linalool was the predominant molecule in the AOs extracted via the steam distillation and microwave–ultrasonic extraction techniques (22.21% and 21.91% (*v*/*v*), respectively) followed by the AOs extracted with the supercritical CO_2_ and hydrodistillation extraction methods (17.7 and 16.87% (*v*/*v*), respectively). In addition, the oxygenated monoterpenoid was the predominant phytochemical group in the AOs from the *C. flexuosus* plant extracted via the microwave–ultrasonic, supercritical CO_2_, steam distillation, and hydrodistillation extraction techniques, which represented 91.94, 91.94, 91.92, and 91.01% (*v*/*v*), respectively.

These outcomes are consistent with the Gao et al. study in which they performed a compositional analysis of *C. flexuosus* AO from China utilizing a GC–MS apparatus and recognized nineteen compounds that made up approximately 99.6% of the total AO. This qualitative analysis revealed that the most abundant compounds were neral (30.4%) and geranial (29.4%) [38].

An investigation performed by Vinutha and Thara Saraswathi characterized 39 compounds from the aerial parts of the *C. flexuosus* plant AO from India that were extracted via the hydrodistillation technique and found that this method gave an oil yield of 1.27% (*v*/*w*). The major compounds of the oxygenated monoterpenoid were citral, 1,7-octadien-3-ol, dimethyl oxatricyclo nonanone, nerol, verbenol, and caryophyllene oxide, representing 64.98, 10.97, 9.44, 2.85, 1.77, and 0.71%, respectively [39].

A study conducted in Tunisia found that the AO obtained from *C. flexuosus* was rich in citral, which represented 85% of the extracted mass and was presented in both cis-isomer (neral) and trans-isomer (geranial) (respectively at 38.89% and 45.86%) [40].

Neral (35.1%) and geranial (44.3%) were the two predominant compounds in the AO obtained from the *C. flexuosus* AO from India extracted with the hydrodistillation method, according to Rajeswara Rao et al. [41]. They also reported that 37 molecules, constituting 80.9% of the oil, were identified in the investigated AO.

These results confirmed that the chemical components and variations in AOs differ significantly from one region to another, reflecting the unique environmental factors, plant species, and cultivation methods specific to each geographical area in addition to the used AO extraction methods [23].

### 3.3. Antifungal Effect

The emergence of antibiotic-resistant fungal pathogens has rendered most available antifungal medications ineffective. Alternative medications are, therefore, required to counteract drug-resistant fungal infections. Combination therapies with AOs and conventional drugs to enhance their efficacy appear to be the most effective solution [42,43]. In this study, the antifungal effect of the AOs from the *C. flexuosus* plant extracted via the microwave–ultrasonic, supercritical CO_2_, steam distillation, and hydrodistillation extraction techniques was evaluated for the first time against some well-known multidrug-resistant fungal strains, namely *C. albicans, C. parapsilosis, C. tropicalis, C. glabrata, T. rubrum, C. auris, B. dermatitidis*, and *H. capsulatum.*

The antifungal screening results revealed that the *C. flexuosus* AOs have potential antifungal effects against all the screened fungi species. The antifungal effects of the AOs extracted with the steam distillation and microwave–ultrasonic methods were remarkable compared with those of the commercial antifungal drug Fluconazole. However, the antifungal effects of the AOs extracted with the hydrodistillation and supercritical CO_2_ methods were a little different against some fungal strains and much weaker than Fluconazole and the AOs extracted with the steam distillation and microwave–ultrasonic methods. Among all the extracted oils, the *C. flexuosus* AO extracted utilizing the steam distillation method exhibits the highest antifungal activity among the other AOs against ATCC *C. albicans* with a MIC of 3.13 ± 0.01 µg/mL compared with Fluconazole, which has a MIC of 1.56 ± 0.03 µg/mL, while the *C. flexuosus* AOs extracted with the steam distillation and microwave–ultrasonic methods were twofold less effective than Fluconazole against the *C. albicans* clinical strains (MIC = 6.25 ± 0.91, 6.25 ± 0.91, and 3.13 ± 0.01 µg/mL, respectively). Moreover, the *C. flexuosus* AOs extracted with these two methods have a more potent antifungal effect against *C. parapsilosis* than Fluconazole with MICs of 3.13 ± 0.01, 3.13 ± 0.01, and 6.25 ± 0.91 µg/mL, respectively, and the same effects were against *T. rubrum* with MICs of 6.25 ± 0.91, 6.25 ± 0.91, and 12.50 ± 0.33 µg/mL, respectively. In addition, the *C. flexuosus* AOs obtained with the steam distillation and microwave–ultrasonic methods have a similar anticandidal effect as Fluconazole on *C. tropicalis* (MICs = 6.25 ± 0.91 µg/mL, respectively), *C. glabrata* (MICs = 3.13 ± 1.01 µg/mL, respectively), *C. auris* (MICs = 25 ± 1.15, respectively), *B. dermatitidis* (MICs = 25 ± 1.15 µg/mL, respectively), and *H. capsulatum* (MICs = 12.5 ± 0.33 µg/mL, respectively).

This is consistent with a previous study established by Gao et al., which found that *C. flexuosus* AO suppressed the growth of *C. albicans* and *C. tropicalis* with MICs of 0.0781% and 0.039%, respectively, while the pure citral molecules had MICs against *C. albicans* and *C. tropicalis* of 0.0313% and 0.0156%, respectively [38]. An investigation established by Al-Ghanayem found that *C. flexuosus* AO at 50 µL/mL concentration has the highest antifungal activity against *C. albicans* with an inhibition zone of 11.33 mm [44]. These differentiations in *C. flexuosus* AO antifungal effects may be due to the varying phytochemical constituents present in the plant and its botanical origin.

The penetration or attachment of natural substances, including AOs, via the fungal membranes can lead to the cell structures disintegrating, making the fungal cells more permeable to the substances [45]. Oxygenated terpenoids have delocalized electrons and may reduce the pH gradient across the cytoplasmic membrane by acting as proton exchangers. The collapse of the proton motive force and the depletion of the ATP pool that result from such an effect can result in the leakage of iron and intracellular cell components and, ultimately, cause cell demise. Terpenoids also exert antifungal activity at the cell membrane level. It has been reported that terpenoids disrupt membrane permeability by altering the fatty acid composition in the membrane, resulting in the loss of the contents of cells [46,47,48].

The differences in the chemical components between the AOs extracted via the steam distillation and microwave–ultrasonic methods and the AOs obtained via the hydrodistillation and supercritical CO_2_ methods made the difference in the antifungal effect. The AOs that are extracted with the steam distillation and microwave–ultrasonic methods have more linalool, which is a powerful antifungal agent that also affects the integrity of the fungi’s plasma membrane and cell wall. Previous investigations revealed the possibility of linalool interacting with three vital enzymes, namely Δ14-sterol reductase, lanosterol 14α-demethylase, and 1,3-β-glucan synthase [49]. Also, the AOs that were extracted with the steam distillation and microwave–ultrasonic methods had more linalyl acetate than the AOs that were extracted with other methods. This added to the possibility that they could suppress the tested fungi growth. This issue agreed with the Singh et al. study, which demonstrated that linalyl acetate enhanced the antifungal effect of the plant’s AO [50]. One of the major extracted *C. flexuosus* AO components was citral, which has already been shown to have antifungal efficacy against molds and yeasts in a variety of environments. Citral has recently been shown to have the capacity to compromise the integrity of the cell membrane, releasing the cellular components of fungal cells and severely restricting their growth through a mechanism of cell membrane damage [51,52,53].

## 4. Materials and Methods

### 4.1. Herbal Material

The aerial parts of *C. flexuosus* including the leaves and stems were harvested from the Tubas area of Palestine. Botanical identification and depositing were performed at An-Najah National University in the Herbal Product Laboratory by pharmacognosist Dr. Nidal Jaradat under the authorization specimen number Pharm-PCT-2784. The aerial parts were thoroughly cleaned before being dried in the shade at ambient temperature (25 ± 2 °C) and relative humidity (55 ± 3 RH). The plant material was then finely pulverized and stored in airtight jars with adequate labeling for further use. The EO yields were expressed in % (*w*/*w*) of the dry material using the following equation: % Yield of the AO = (weight of the extracted AO (g)/weight of the dried plant material (g)) × 100.

### 4.2. Aromatic Oils Extraction Assays

#### 4.2.1. Hydrodistillation Extraction Method

The AO of the *C. flexuosus* plant was extracted utilizing the simple water-distillation procedure [54]. Briefly, 0.5 kg of the dried aerial parts’ powder was suspended with 0.5 L of deionized water, and the EO was extracted using a hydrodistillation technique connected with the Clevenger apparatus operating at atmospheric pressure for 180 min at 100 °C. The extracted AO was chemically dried using magnesium sulfate and stored at 2–6 °C until further use.

#### 4.2.2. Microwave–Ultrasonic Extraction Method

In this method, the AOs were separated using a microwave oven with ultra-sonication. To enhance the extraction procedure, ultrasonic waves were applied to the plant powder aqueous solution. The Ultrasonic–microwave Cooperative Extractor/Reactor (CW-2000, Xi’an, China), which combines an ultrasonic extractor and a microwave oven, was employed in this investigation. This equipment was filled with a one L round-bottom flask holding 0.5 kg of the plant. The plant was suspended in approximately 0.5 L of deionized water in this flask. The Clevenger apparatus was then inserted into the same setup and attached to the flask. The power of the microwave–ultrasonic extractor device was set at 1000 W while the extraction procedure was being performed. The device’s ultrasonic power was set to 50 W at a frequency of 40 kHz, which is its highest power setting. This device was used to extract material for 10 min at 100 °C [55]. The resulting AO was extracted into a spotless beaker, chemically dried utilizing magnesium sulfate, and kept in tightly covered dark amber bottles in a 2–6 °C refrigerator.

#### 4.2.3. Supercritical CO_2_ Extraction Method

According to Elsayed and co-workers’ investigation, the supercritical fluid CO_2_ extractor (Delta Electronics Inc., VFD022M43B, Shanghai, China) connected with an industrial chiller (Shen Zhen DA Inc., IC150-T/TNTH, Shenzhen, China) and supercritical CO_2_ with a purity of more than 99.98% were utilized for the separation of *C. flexuosus* AO [56]. The air-dried, powdered *C. flexuosus* plant material (0.5 kg) was placed in an extractor stainless steel vessel, the temperature was adjusted to 40 °C, and the pressure was fixed at 15.0 MPa. The flow rate of CO_2_ was 0.2 kg CO_2_/h. The AO was completely extracted from the plant material after 15 min and then dried using magnesium sulfate. The extracted AO was kept in an amber bottle in a refrigerator at 2–6 °C.

#### 4.2.4. Steam Distillation Extraction Method

To extract the AO with steam distillation, the powdered *C. flexuosus* plant material was placed in a round-bottom flask, which was connected to the lower flask containing water and from the upper part connected with the Clevenger apparatus. This procedure was established at atmospheric pressure for 240 min at 100 °C. The water vapor produced in the flask crosses the herb material, is charged with AO, and then moves to the Clevenger apparatus where it is condensed. After condensation, the AO is separated from the water with decantation, and then, the AO is dried using magnesium sulfate and kept in an amber bottle in a refrigerator at 2–6 °C [57].

### 4.3. GC–MS Analysis

To qualitatively and quantitatively estimate the AO of the *C. flexuosus* plant extracted by using the four different extraction methods, gas chromatography–mass spectrometry (GC–MS) chromatograms were recorded utilizing a Shimadzu (GC–MS Shimadzu, QP-5000, Tokyo, Japan) machine. An Rtx-5 ms column (30 m long, 0.25 mm thickness, and 0.250 mm internal diameter) was set up in the GC. The injector temperature was 220 °C. The oven’s temperature was controlled by raising it from 50 °C (1 min hold) to 130 °C and then to 250 °C and maintaining it isothermally for 15 min. Helium was employed as a carrier gas at a flow rate of 1 mL/min. The transfer line temperature was 290 °C. An electron ionization system with a 1.7 KV detector voltage was employed for GC–MS detection. A mass range of 38–450 M/Z was covered, employing a 0.5 s scan rate and a scan speed of 1000 amu/s. The phytochemical constituents of the AO were recognized by matching their Kovats’ retention indices (RI) with those of the *n*-alkanes standard series (C_8_–C_28_). In addition, their mass spectra were compared with those reported in the Wiley mass spectral database and the National Institute of Standards and Technology (NIST08 and NIST11) (similarity index > 90%) [58].

### 4.4. Antifungal Activity

For the assessments of the antifungal activity of the *C. flexuosus* AOs’ minimum inhibitory concentrations (MICs), the broth microdilution method was used. In brief, eight clinically confirmed fungal isolates, *Candida parapsilosis* (CC-145), *Candida tropicalis* (CC-154), *Candida albicans* (CC-201), *Candida glabrata* (204), *Trichophyton rubrum* (210), *Candida auris* (CC-240), *Blastomyces dermatitidis* (CC-253), and *Histoplasma capsulatum* (CC-261), were used in addition to a reference laboratory *Candida albicans* strain (ATCC 90028). Briefly, DMSO was used to solubilize the AOs to a concentration of 200 µg/mL. From the resulting solution, a two-fold serial microdilution was performed 10 times in sterile brain heart infusion broth (BHIB) media. The dilutions were carried out in 96-well disposable plastic trays in aseptic conditions; these 10 wells will contain a gradient of concentrations of the EOs mixed with a standard fungal amount. The other two remaining wells were used as controls; one was a positive growth control that contained media and microbes only, while the other was a negative growth control that contained media alone. The microwell plates were then incubated at 35 °C for 24–48 h. The MIC was approved to be the lowest concentration that shows no fungal growth. To evaluate the whole procedure, Fluconazole was used as an antifungal activity-positive control [59].

### 4.5. Statistical Analysis

All established tests were performed in triplicate. The results were expressed as the means (±) standard deviation (SD), representing the average value along with the variability in the data. The statistical analysis was carried out, and a *p*-value less than 0.001 was considered statistically significant.

## 5. Conclusions

The supercritical CO_2_ method demonstrated its supremacy by producing the highest AO yield followed closely by the microwave–ultrasonic and steam distillation methods. On the contrary, the hydrodistillation approach produced the lowest oil yield. The chemical composition analysis of the AOs revealed a consistent profile across the different extraction techniques with the majority of key molecules being present in high percentages. Remarkably, the extracted AOs exhibited substantial antifungal properties against a range of fungi. Notably, the AOs obtained with the steam distillation and microwave–ultrasonic methods displayed particularly potent antifungal effects, even surpassing the performance of the commercial antifungal drug Fluconazole in certain cases. Notably, *C. parapsilosis* and *T. rubrum*, two common fungi strains, were notably susceptible to the AOs extracted via these methods. These findings underscore the significance of the extraction techniques in determining the chemical composition and biological activities of *C. flexuosus* AO. Moreover, the observed antifungal efficacy against clinically relevant fungi species suggests the potential applicability of these AOs as natural antifungal agents. Further research is warranted to delve deeper into the specific mechanisms underlying these effects and explore potential applications in the pharmaceutical, food, and cosmetic industries.

## Figures and Tables

**Table 1:** Phytochemical components (% (*v*/*v*)), retention time, retention indexes, and yields (*w*/*w*) of *C. flexuosus* AOs extracted with hydrodistillation, steam distillation, microwave–ultrasonic, and supercritical CO_2_ extraction procedures.

Names	Retention Time	Retention Index	Reference Retention Index	Microwave–Ultrasonic Method % (*v*/*v*) of AO	Steam Distillation Method % (*v*/*v*) of AO	Hydrodistillation Method % (*v*/*v*) of AO	Supercritical CO_2_ Method % (*v*/*v*) of AO
Camphene	10.9	955	951	0.29	0.3	0.34	0.37
Methyl heptenone	12.39	988	986	1.31	1.38	1.44	1.4
β-Myrcene	12.56	992	993	1.34	0.88	2.12	2.32
Hexyl acetate	13.51	1013	1015	0.48	0.51	0.37	0.35
Limonene	14.28	1030	1030	1.71	1.76	1.71	1.69
Eucalyptol	14.42	1033	1033	1.39	1.35	1.11	1.21
Cis-β-ocimene	14.64	1038	1040	0.18	0.18	0.14	0.1
Linalool	17.26	1102	1105	21.91	22.21	16.87	17.7
Camphor	19.25	1149	1148	1.16	1.16	0.88	0.9
Citronellal	19.55	1156	1156	0.43	0.47	0.51	0.43
Borneol	20.23	1172	1172	0.41	0.43	0.37	0.38
Terpinen-4-ol	20.7	1183	1180	0.59	0.6	0.45	0.47
α-Terpineol	21.3	1197	1198	2.55	2.53	1.98	2.13
Neral (β-citral)	22.87	1246	1248	17.41	16.93	22.09	21.49
Linalyl acetate	23.34	1258	1257	24.61	24.34	18.77	20.86
Geranial (α-citral)	24.00	1275	1278	20.64	20.96	27.01	25.6
Geranyl acetate	27.29	1373	1383	0.84	0.94	0.97	0.77
Isocaryophyllene	28.14	1404	1405	1.35	1.28	1.18	1.19
**Total**				**98.6%**	**98.21%**	**98.31%**	**99.36%**
**Yields**				**1.55± 0.05** (*w*/*w*)	**1.24 ± 0.04** (*w*/*w*)	**1.17 ± 0.01** (*w*/*w*)	**1.62 ± 0.03** (*w*/*w*)
	**Phytochemical Groups**						
**Hydrocarbon monoterpene**				3.52	3.12	4.31	4.48
**Oxygenated monoterpenoid**				91.94	91.92	91.01	91.94
**Hydrocarbon sesquiterpene**				1.35	1.28	1.18	1.19
**Others**				1.79	1.89	1.81	1.75
**Total**				98.6	98.21	98.31	99.36

**Table 2:** Comparison of the minimum inhibitory concentrations (μg/mL) of the AOs from *C. flexuosus* extracted via hydrodistillation, steam distillation, microwave–ultrasonic, and supercritical CO_2_ extraction methods to those of the reference medication Fluconazole (μg/mL).

	Fungus								
Source of Fungi	ATCC	Clinical Species							
Assigned name/number	90028	145	154	201	204	210	240	253	261
**Microbe**	***Candida albicans***	***Candida parapsilosis***	***Candida tropicalis***	***Candida albicans***	***Candida glabrata***	***Trichophyton rubrum***	***Candida auris***	***Blastomyces dermatitidis***	***Histoplasma capsulatum***
AO extracted with hydrodistillation method	12.5 ± 0.33	50 ± 1.24	12.5 ± 0.33	12.5 ± 0.33	6.25 ± 0.91	25 ± 1.15	50 ± 1.24	50 ± 1.24	25 ± 1.15
AO extracted with steam distillation method	3.13 ± 0.01	3.13 ± 0.01	6.25 ± 0.91	6.25 ± 0.91	3.13 ± 0.01	6.25 ± 0.91	25 ± 1.15	25 ± 1.15	12.5 ± 0.33
AO extracted with microwave–ultrasonic method	6.25 ± 0.91	3.13 ± 0.01	6.25± 0.91	6.25 ± 0.91	3.13 ± 0.01	6.25 ± 0.91	25 ± 1.15	25 ± 1.15	12.5 ± 0.33
AO extracted with supercritical CO_2_ method	25 ± 1.15	25 ± 1.15	12.5 ± 0.33	25 ± 1.15	12.5 ± 0.33	25 ± 1.15	50 ± 1.24	50 ± 1.24	25 ± 1.15
Fluconazole	1.56 ± 0.03	6.25 ± 0.91	6.25 ± 0.91	3.13 ± 0.01	3.13 ± 0.01	12.5 ± 0.33	25 ± 1.15	25 ± 1.15	12.5 ± 0.33

Results were expressed as mean (*n* = 3) with ± SD values (*p* < 0.001).

## Data Availability

Not applicable.
